# Data traceability and interpretational limits in comparing child maltreatment forms and depression

**DOI:** 10.1017/S0033291726104966

**Published:** 2026-07-22

**Authors:** Shuai Gao, Qi-long Li, Pei-long Li

**Affiliations:** Department of Pediatrics, Henan Provincial People’s Hospital, Zhengzhou University People’s Hospital, Zhengzhou, China

## To the Editor

We carefully reviewed the multilevel meta-analysis by Rind and Rieger (2026) on the association between five types of childhood abuse and depression. The authors’ effort to improve comparability across abuse types through a ‘complete-abuse’ design is valuable; however, we identified at least two issues that require clarification and that, in our view, are sufficient to call into question the study’s central conclusion that ‘emotional abuse has the strongest association, while sexual abuse has the weakest’.

First, in our targeted review of the supplementary source dataset and the cited primary studies, we found that the Supplementary Materials classify Hovens et al. (2015) as a ‘risk study’ and report the following coefficients: EA = −0.58, PA = 0.85, and SA = 0.56 (*n* = 1,167). However, Hovens et al. did not report results using the EA/PA/SA tripartite classification. Instead, the original study assessed emotional neglect, psychological abuse, physical abuse, and sexual abuse. Thus, the EA value reported in the Supplementary Materials does not fully correspond to the original study’s variables, at least at the level of nomenclature, and appears to align more closely with the original category of ‘psychological abuse’. More importantly, the original article does not support the interpretation of this variable as having a negative or protective effect. On the contrary, the results section explicitly states that each trauma domain is associated with the subsequent onset of depressive, anxiety, and comorbid disorders, and psychological abuse is not reported as showing an inverse association. Accordingly, the entry ‘EA = −0.58’ lacks a clear explanation of variable mapping and makes it difficult to trace a consistent statistical meaning back to the original study.

Second, although the authors state that duplicate studies were removed, the ‘risk studies’ section of the Supplementary Materials includes both Harrop-Griffiths et al. (1988) and Walker et al. (1988). These studies share highly similar author groups, publication years, and titles, and their publicly available abstracts appear to describe the same University of Washington sample: 25 women with chronic pelvic pain and 30 controls, for a total of 55 participants. If both studies were included simultaneously, the weighting assigned to certain abuse categories would be directly affected. Given the authors’ emphasis on constructing a more comparable database after deduplication, we believe they should publicly specify their deduplication criteria and report sensitivity analyses conducted after excluding potentially duplicate samples.

More importantly, the interpretation of the ‘complete-abuse’ results appears overly strong. The main advantage of the complete-abuse design is that different abuse types are measured within the same cohort, thereby reducing confounding arising from differences between samples. However, this design does not address the fact that the same individual often experiences multiple forms of abuse simultaneously (Edwards et al., 2003). Indeed, the Supplementary Materials show that the various abuse types are highly correlated within studies. Consequently, the rankings presented in the article seem to reflect comparisons of the overall associations between different forms of abuse and depression, rather than comparisons of their independent effects. Put differently, these findings do not demonstrate that emotional abuse itself is necessarily more likely to cause depression than other forms of abuse. Furthermore, many of the original studies relied on retrospective self-report measures, and items assessing emotional abuse or neglect are conceptually closer to depressive symptoms, such as low self-worth and feelings of rejection. This conceptual overlap may inflate the observed association with depression. Therefore, the hierarchical differences reported by the authors may reflect not only genuine variation in risk but also the effects of co-occurring abuse and common method bias.

We therefore recommend, at a minimum, that the authors supplement their analysis with a cross-validation of data-processing procedures and conduct sensitivity analyses after excluding questionable entries. Until these issues are resolved, it is premature to characterize the study as ‘providing clearer evidence that emotional abuse is the strongest’.

## Data Availability

The data underlying this article are derived from published studies and are available in the public domain.
